# Inter-limb differences in upper quarter mobility/stability are not associated with performance in competitive swimmers

**DOI:** 10.3389/fspor.2024.1382779

**Published:** 2024-04-08

**Authors:** Katharina Borgmann, Stefan Panzer, Sam Limpach, Thomas Muehlbauer

**Affiliations:** ^1^ Division of Movement and Training Sciences/Biomechanics of Sport, University of Duisburg-Essen, Essen, Germany; ^2^ Institute of Sport Science, Saarland University, Saarbrücken, Germany

**Keywords:** shoulder function, Y Balance Test–Upper Quarter, distance reaching, reach asymmetry, side difference, athletes

## Abstract

**Background:**

The Y Balance Test–Upper Quarter (YBT–UQ) is a cost-effective, well-established, closed kinetic chain test to assess inter-limb asymmetries in the upper quarter that could negatively affect swimming performance. Thus, the aim of the present study was to determine YBT–UQ performances and inter-limb differences as well as its association with swimming performance in athletes with diverging levels of expertise.

**Methods:**

Forty female and male competitive swimmers (age range: 10–22 years) with different expertise levels (A-squad: *n *= 9, B-squad: *n *= 12, C-squad: *n *= 19) were tested (reach distances for the YBT–UQ) and swimming performance was calculated using the ratio of individual to world best time.

**Results:**

YBT–UQ performances (i.e., inferolateral reach direction for the dominant arm: *p *= .027, *η_p_*^2 ^= .12 and the non-dominant arm: *p *= .031, *η_p_*^2 ^= .17) but not YBT–UQ inter-limb differences significantly differed between groups and were largest in swimmers with the lowest expertise level (i.e., C-squad). Further, YBT–UQ performances (i.e., inferolateral reach direction [*r *= −.68 to −.70, both *p *< .05] and composite score [*r *= −.65 to −.67, both *p *< .05] for both arms and medial reach direction for the non-dominant arm [*r *= −.64, *p *< .05]) but not inter-limb differences were significantly and negatively correlated with swimming performance among B-squad swimmers.

**Conclusions:**

Our results suggest that inter-limb differences in upper quarter mobility/stability are not influenced by the level of expertise and have no significant associations with swimming performance. However, greater reach distances were correlated with lower swimming performance for the B-squad swimmers indicating that a training-related increase in upper quarter mobility/stability could worsen swimming performance in those athletes.

## 1 Introduction

The Y Balance Test–Upper Quarter (YBT–UQ) is a well-established, cost-effective, and frequently used field-based method to investigate upper quarter mobility/stability in athletes performing overhead actions (1, 2). Particular in swimming, the assessment of upper quarter mobility/stability is important from both a health-related and a performance-related perspective. In the first case, there are studies that reported a large number of injuries (3) and an increased risk of injury (4) for the shoulder region in swimmers. In the second case, studies have shown differences in YBT–UQ performance depending on swimmers' competition level (5) as well as significant associations between upper quarter mobility/stability and swimming performance (6). Specifically, Bullock et al. (5) showed significantly better YBT–UQ performances in collegiate swimmers (*N *= 70; 20.8 ± 1.2 years) in comparison to high school swimmers (*N *= 70; 17.0 ± 1.1 years) for the medial and inferolateral reach directions. Further, Bartolomeu et al. (6) reported significant moderate positive correlations between YBT–UQ performance and swimming speed, i.e., swimmers (*N *= 16; ≈20.0 ± 2.0 years) who had a large reach distance achieved a faster swimming speed.

Usually, the YBT–UQ is recorded for both sides, thus inter-limb differences (i.e., asymmetry) can be detected as well as their association with athletic performance (7). In the first case, most of the available studies did not detect side differences in swimming (5, 8, 9) or in other sports like baseball (10, 11) and softball (10, 12). However, these studies were almost exclusively performed with adult athletes, which limits the transferability of the findings to young athletes due to growth, maturation, and development processes (13). In the second case, only one study (6) exists to date, which analysed the relationship between swimming performance and inter-limb asymmetry in upper quarter mobility/stability using a dry-land test (i.e., YBT–UQ). The authors showed no significant correlations between asymmetry value and swimming speed. However, this finding is again limited to adult swimmers performing at a recreational level. Thus, it remains unclear whether the relationship between inter-limb asymmetry in upper quarter mobility/stability and swimming performance is affected by different performance levels. In other words, swimmers with a low vs. high level of expertise may have greater inter-limb differences in upper quarter mobility/stability, which may lead to negative associations with swimming performance.

Thus, the present study aimed to investigate inter-limb differences in upper quarter mobility/stability and their association with swimming performance in competitive swimmers with diverging levels of expertise (i.e., A-, B-, and C-squad). We hypothesised that due to the several years of experience required for the development of movement symmetry, inter-limb differences in upper quarter mobility/stability would be particularly present in swimmers with a low level of expertise, decrease with advancing expertise, and are negatively associated with swimming performance.

## 2 Material and methods

### 2.1 Participants

An *a priori* power analysis with G*Power, version 3.1.9.7 (14) showed that a total of 37 participants would be required. The analysis was run with *ρ *= 0.40, *α *= 0.05, 1-*β *= 0.80. A total of 40 competitive swimmers with different levels of expertise participated in this cross-sectional study after experimental procedures were explained (Table 1). All athletes were free of any musculoskeletal dysfunction, neurological impairment, or orthopaedic pathology within the preceding three months. Participant's assent and written informed consent of the parents or legal guardians were obtained before the start of the study. The Human Ethics Committee at the University of Duisburg-Essen, Faculty of Educational Sciences approved the study protocol.

**Table 1:** Characteristics of the swimmers (*N *= 40) by level of expertise.

Characteristic	A-squad (*n *= 9)	B-squad (*n *= 12)	C-squad (*n *= 19)	*p*-value (*η*_p_^2^)
Age [years]	17.1 ± 2.3	13.3 ± 0.9	10.6 ± 0.5	**<**.**001 (.83)**
Training experience [years]	10.2 ± 1.9	6.8 ± 2.1	4.7 ± 1.1	**<**.**001 (.67)**
Training volume [min/wk]	1,270.0 ± 140.7	1,043.8 ± 130.4	743.7 ± 182.4	**<**.**001 (.66)**
Preferred style [BK/BR/FL/FR]	1/1/1/6	0/5/0/7	2/2/3/12	–
Sex [f/m]	5/4	6/6	12/7	–
Body height [cm]	178.9 ± 7.8	168.3 ± 13.4	150.0 ± 6.7	**<**.**001 (.64)**
Body mass [kg]	70.1 ± 8.2	55.6 ± 14.6	39.0 ± 5.6	**<**.**001 (.64)**
Body mass index [kg/m^2^]	22.0 ± 1.2	19.2 ± 2.4	17.2 ± 1.4	**<**.**001 (.56)**
Dominant arm [L/r]	1/8	0/12	5/14	**–**
Dominant arm length [cm]	92.7 ± 6.0	86.6 ± 8.0	75.8 ± 4.3	**<**.**001 (.60)**
Non-dominant arm length [cm]	92.7 ± 6.5	86.1 ± 7.9	75.9 ± 4.3	**<**.**001 (.59)**

Values are expressed as mean ± standard deviation. Figures in brackets are effect sizes (*η_p_*^2^) with .02 ≤ *η_p_*^2 ^≤ .12 indicating small, .13 ≤ *η_p_*^2 ^≤ .25 indicating medium, and *η_p_*^2 ^≥ .26 indicating large effects. Bold values indicate a statistically significant difference (*p* < .05). BK, backstroke; BR, breaststroke; FL, butterfly; FR, freestyle; f, female; l, left; m, male; r, right.

### 2.2 Procedure

The experimental procedure included the assessment of anthropometric variables followed by the assessment of upper quarter mobility/stability. Both were explained using standardised verbal instructions and a visual demonstration.

### 2.3 Assessments

#### 2.3.1 Assessment of anthropometric variables

Body mass was assessed using an electronic scale (Seca 803, Basel, Switzerland) to the nearest 100 g, with participants wearing light clothing but no shoes. Standing body height was measured with a stadiometer (Seca 217, Basel, Switzerland) to the nearest 0.1 cm, with participants standing straight and upright without shoes. Arm length (AL) was determined to the nearest 0.1 cm from the distal tip of the middle finger, with the shoulder at 90-degree abduction, to the seventh cervical spinous process using a measuring tape (15).

#### 2.3.2 Assessment of upper quarter mobility/stability

The YBT–UQ was performed using a commercially available Y Balance Test Kit (Move2Perform, Evansville, IN, United States). All participants had to start in a single arm push-up position (15) with the third metacarpophalangeal joint in the centre of the test kit (8) and their feet shoulder width apart. The right arm was always the first stance arm, and the mobile reach indicator had to be moved by the left arm in the medial, inferolateral, and superolateral reach directions in a continuous manner, without rest between movements. After six trials (i.e., 3 practice trials followed by 3 data-collection trials separated by a 30-s rest period) with the right arm as the stance arm, the same procedure was repeated with the left arm as the stance arm and the right arm as the reach arm. The between-trials reliability of the YBT–UQ ranged from ICC = 0.78–0.94 for the different reach directions. A three-point contact (both feet on the floor and the stance arm on the test kit) had to be maintained throughout each trial. This procedure was visually monitored by the examiner (i.e., graduated sport scientist) and was valid if participants (1) maintained the single arm push-up position (i.e., did not touch the floor with the reach arm), (2) remained in contact with the reach indicator at the most distal point (i.e., did not push the reach indicator to achieve greater distance), (3) did not use the reach indicator to support weight (i.e., mechanical support), and (4) returned the reach arm to the centre of the test kit. The participants had a 30-s rest period between right and left arm trials. Only the best score (i.e., trial with the largest reach distance in cm) for each reach direction was used for further analyses (15). Reach distance was normalised to AL [i.e., (reach distance/AL) × 100]) and a normalized composite score was calculated (i.e., [(medial + inferolateral + superolateral)/(3 × AL)] × 100) (16). Further, the limb symmetry index (LSI) was calculated as the absolute value of the ratio between the non-dominant and the dominant arm (17). Validity as well as reliability (i.e., “moderate-to-good” to “excellent” ICC values) of the YBT–UQ has been shown in previous studies (18, 19).

#### 2.3.3 Estimation of swimming performance

The estimation of swimming performance was based on a points table obtained from World Aquatics [until 2022 Fédération Internationale de Natation (FINA)]. Specifically, an individual age- and sex-specific point value (*p*) was calculated, with B representing the base time (i.e., current world record) and T the individual time achieved in the preferred swimming style and distance. The corresponding formula is: *p* = 1,000 × (B/T)^3^.

### 2.4 Statistical analyses

Data were analysed using SPSS version 28.0 (IBM Inc., Chicago, IL) and are presented as mean values ± standard deviations. After normal distribution (Shapiro-Wilk test) and homogeneity of within variance/sphericity (Levene test) was not rejected, an analysis of variance (ANOVA) was performed to detect differences between squads. Bonferroni-adjusted post-hoc analyses were performed if a significant difference occurred. Partial eta-squared (*η_p_*^2^) was calculated and reported as small (.02 ≤ *η_p_*^2 ^≤ .12), medium (.13 ≤ *η_p_*^2 ^≤ . 25), or large (*η_p_*^2 ^≥ .26) for the ANOVA (20). Further, associations between YBT-UQ inter-limb difference and swimming performance were separately calculated for each squad using Pearson's product moment correlation coefficient. Coefficients were interpreted as weak (*r *= .10–.35), moderate (*r *= .36–.67), or strong (*r *= .68–1.00) (21). The alpha value was *a priori* set at *p *< .05 for all analyses.

## 3 Results

### 3.1 YBT–UQ inter-limb difference/performance and swimming performance by expertise level

The ANOVA showed no significant group differences for the LSI values and only a few significant differences for the YBT–UQ reach distances (Table 2). Precisely, significant small to medium group differences were only found for the inferolateral reach direction in the dominant (*p *= .027, *η_p_*^2 ^= .12) and non-dominant (*p *= .031, *η_p_*^2 ^= .17) arm. Post-hoc analyses revealed significantly larger reach distances for the C-squad compared to the A-squad (dominant arm: *p *= .036; non-dominant arm: *p *= .032) but not to the B-squad (dominant arm: *p *= .218; non-dominant arm: *p *= .397) swimmers. Further, swimming performance significantly differed between groups (*p *< .001, *η_p_*^2 ^= .82) and post-hoc analyses yielded higher values for the A-squad compared to the B-squad (*p *< .001) and C-squad (*p *< .001) swimmers as well as the B-squad compared to the C-squad swimmers (*p *< .001).

**Table 2:** Y Balance Test–Upper Quarter inter-limb difference/performance and swimming performance by level of expertise.

Outcome	A-squad (*n *= 9)	B-squad (*n *= 12)	C-squad (*n *= 19)	*p*-value (*η*_p_^2^)
YBT–UQ: inter-limb difference				
LSI for the medial reach [% AL]	4.0 ± 2.5	3.6 ± 2.4	4.2 ± 4.0	.891 (.01)
LSI for the inferolateral reach [% AL]	6.1 ± 5.0	7.0 ± 6.0	6.1 ± 4.9	.887 (.01)
LSI for the superolateral reach [% AL]	4.3 ± 3.1	7.6 ± 7.4	7.5 ± 5.4	.339 (.06)
LSI for the composite score [% AL]	3.4 ± 2.5	3.8 ± 4.7	4.1 ± 2.6	.843 (.01)
YBT–UQ: performance				
DA: medial reach [% AL]	102.1 ± 5.7	102.9 ± 7.1	101.9 ± 7.2	.920 (.01)
DA: inferolateral reach [% AL]	93.5 ± 15.3	98.1 ± 11.2	106.2 ± 10.5	.**027** (**.12)**
DA: superolateral reach [% AL]	75.0 ± 6.0	77.9 ± 10.1	81.1 ± 9.4	.249 (.07)
DA: composite score [% AL]	90.2 ± 7.0	93.0 ± 8.3	97.4 ± 9.1	.102 (.12)
NDA: medial reach [% AL]	102.6 ± 7.1	101.9 ± 7.8	104.9 ± 9.5	.586 (.03)
NDA: inferolateral reach [% AL]	93.1 ± 15.3	99.6 ± 11.1	106.7 ± 11.9	.**031** (**.17)**
NDA: superolateral reach [% AL]	77.7 ± 5.6	82.8 ± 10.3	85.4 ± 9.0	.111 (.11)
NDA: composite score [% AL]	91.1 ± 6.9	94.7 ± 7.8	98.0 ± 8.0	.095 (.12)
Swimming performance				
Value [pt.]	661.3 ± 68.2	516.6 ± 42.2	301.6 ± 85.8	**<**.**001 (.82)**

Values are expressed as mean ± standard deviation. Figures in brackets are effect sizes (*η_p_*^2^) with .02 ≤ *η_p_*^2 ^≤ .12 indicating small, .13 ≤ *η_p_*^2 ^≤ .25 indicating medium, and *η_p_*^2 ^≥ .26 indicating large effects. Bold values indicate a statistically significant difference (*p* < .05). AL, arm length; DA, dominant arm; LSI, limb symmetry index; NDA, non-dominant arm; YBT–UQ, Y Balance Test–Upper Quarter.

### 3.2 Correlations between YBT–UQ inter-limb difference/performance and swimming performance by expertise level

Non-significant positive and negative correlations were observed between LSI values and swimming performance, regardless of expertise level (Table 3). For the YBT–UQ reach distances, we detected significant inverse correlations for the inferolateral (*r *= −.41, *p *< .01), and the superolateral (*r *= −.33, *p *< .05) reach direction and the composite score (*r *= −.37, *p *< .05) for the dominant arm as well as the inferolateral (*r *= −.44, *p *< .01) and superolateral (*r *= −.33, *p *< .05) reach direction and the composite score (*r *= −.37, *p *< .05) for the non-dominant arm, when considering all swimmers. Regarding group-specific analyses, significant inverse correlations were found for the B-squad but not for the A-squad and C-squad swimmers. Precisely, the inferolateral reach direction (*r *= −.70, *p *< .05) and the composite score (*r *= −.65, *p *< .05) for the dominant arm as well as the medial (*r *= −.64, *p *< .05) and inferolateral (*r *= −.68, *p *< .05) reach direction and the composite score (*r *= −.67, *p *< .05) for the non-dominant arm were significantly negatively correlated with swimming performance in B-squad swimmers only.

**Table 3:** Correlations of Y Balance Test–Upper Quarter inter-limb difference/performance with swimming performance by level of expertise.

Outcome	Swimming performance [pt.]			
	All swimmers (*n *= 9)	A-squad (*n *= 9)	B-squad (*n *= 12)	C-squad (*n *= 19)
YBT–UQ: inter-limb difference				
LSI for the medial reach [% AL]	−.05	−.23	.56	−.08
LSI for the inferolateral reach [% AL]	.03	.09	.06	−.03
LSI for the superolateral reach [% AL]	−.14	−.13	.18	.09
LSI for the composite score [% AL]	−.09	−.29	−.03	.09
YBT–UQ: performance				
DA: medial reach [% AL]	−.02	.27	−.56	−.09
DA: inferolateral reach [% AL]	−.41**	.28	−.70*	−.08
DA: superolateral reach [% AL]	−.33*	−.17	−.43	−.16
DA: composite score [% AL]	−.37*	.23	−.65*	−.13
NDA: medial reach [% AL]	−.15	.13	−.64*	.04
NDA: inferolateral reach [% AL]	−.44**	.19	−.68*	−.23
NDA: superolateral reach [% AL]	−.33*	−.22	−.30	−.03
NDA: composite score [% AL]	−.37*	.12	−.67*	−.08

Values are expressed as Pearson's product moment correlation coefficient with *r *= .10 – .35 indicating weak, *r *= .36–.67 indicating moderate, and *r *= .68–1.00 indicating strong associations [14].

AL, arm length; DA, dominant arm; LSI, limb symmetry index; NDA, von-dominant arm; YBT–UQ, Y Balance Test–Upper Quarter.

* *p *< .05.

** *p *< .01.

## 4 Discussion

In the present study, we investigated inter-limb differences and performance in upper quarter mobility/stability and their relationship with swimming performance in competitive swimmers with different levels of expertise (i.e., A-, B-, and C-squad). Two major findings emerged: (a) YBT–UQ performance (i.e., inferolateral reach direction for both arms) but not inter-limb difference was largest in swimmers with the least expertise (i.e., C-squad); (b) YBT–UQ performance (i.e., inferolateral reach direction and composite score for both arms and medial reach direction for the non-dominant arm) but not inter-limb difference was significantly negatively correlated with swimming performance in B-squad swimmers.

The first part of our hypothesis stating that inter-limb differences in upper quarter mobility/stability would be particularly present in swimmers with a low level of expertise and decrease with advancing expertise was not confirmed. Contrary, we found no significant differences for the LSI values depending on the expertise level. This is consistent with a previous study (5) also stating no statistical differences in reach asymmetries between competition levels (i.e., collegiate vs. high school swimmers). The lack of significant differences in LSI values between the groups can rely on the high variability of inter-limb asymmetries (9), which can be seen in the relatively large standard deviations shown in Table 2. According to Maloney (22), inter-limb asymmetry can vary depending on the practised type of sport, the volume of exposure, and the assessed physical performance, which makes comparative analyses difficult. As a result, it is advisable to repeatedly analyse changes in inter-limb asymmetry within the same population of athletes over a longer period (e.g., over the course of a season). This would make it possible to distinguish between intra-individual and inter-individual characteristics in side differences. Intriguingly, we detected significantly greater YBT–UQ performance for the inferolateral reach direction for both arms in swimmers with the least expertise (i.e., C-squad) compared to those with the largest level (i.e., A-squad). This finding is in contrast to a previous study from Bullock and colleagues (5) who reported significantly better YBT–UQ performance for the inferolateral reach direction in swimmers with a high (collegiate) compared to a low (high school) level of expertise. The significantly greater reach values for the inferolateral direction for both arms in swimmers with the least expertise suggests a better upper quarter mobility/stability for this specific direction. In other words, the ratio of reach distance to arm length was more favourable for this direction. One reason could be that the C-squad swimmers are more capable to combine core stability, scapular stability/mobility (scapular upward rotation), and thoracic rotation (twist movement). Another explanation that we cannot exclude is the possibility of growth and maturation processes during childhood and adolescence (23). These processes occur in a curvilinear rather than a linear fashion (24). That is, changes in YBT–UQ performance vary depending on the stage of growth, maturation, and development, despite the same time periods (25). In this regard, Schwiertz and colleagues (25) showed that, especially for girls, significantly better reach values were achieved for the inferolateral direction by 10–11-year-olds compared to 12–13, 14–15, and 16–17-year-olds. In the present study, significantly better reach values were also achieved by the younger swimmers with the least expertise level (C-squad) and the highest proportion of females (63%). This indicates that age- but also sex-specific normative values are necessary for an adequate classification of YBT-UQ performance in youth athletes. The significantly better YBT–UQ performance of the C-squad compared to the A-squad swimmers was only found for the inferolateral reach direction. Reasons for this direction-specific finding remain unclear to this point and require further investigations. A possible reason might be the relatively high proportion of swimmers (12 out of 19) stating freestyle as their preferred swimming style, which demand the ability to perform scapular upward rotation combined with thoracic rotation (twist movement).

The second part of our hypothesis stating that inter-limb differences in upper quarter mobility/stability are negatively associated with swimming performance was also not confirmed. Consequently, the observed LSI values of ≈ 3.4%–6.1%, ≈ 3.6%–7.6%, and ≈ 4.1%–7.5% in A-, B-, and C-squad swimmers respectively that were below injury-related cut-off values (≥7.75%), did not have a negative effect on swimming performance. This finding supports previous research (6, 9) also reporting no or rarely impact of inter-limb asymmetry on swimming performance. However, we observed negative correlations between YBT–UQ performance (i.e., inferolateral reach direction and composite score for both arms and medial reach direction for the non-dominant arm) and swimming performance in B-squad swimmers. Precisely, greater reach distances for the inferolateral direction and the composite score of both arms as well as for the medial direction of the non-dominant arm were correlated with lower swimming performance. This finding is contrary to those of Bartolomeu et al. (6) reporting positive correlations between the composite score of both arms and swimming speed and indicates that for the B-squad swimmers a training-related increase in upper quarter mobility/stability could worsen their swimming performance. It is not yet clear why only the B-squad swimmers showed significant negative correlations between YBT–UQ and swimming performance. One reason could be that this group can be categorised as adolescent athletes. During this phase (i.e., “adolescence growth spurt”), relatively major physical changes occur, which are typically attributed to growth (i.e., increase in stature, body mass, and body dimensions) and maturation (i.e., somatic, skeletal, and sexual maturity) (26) and can negatively influence performance (27).

The present study has some limitations that should be discussed. Study sample size was determined based on the primary research question (correlation between inter-limb asymmetries in YBT–UQ and swimming performance). Thus, our sample is possibly underpowered to detect between-group differences. Moreover, the assessment of inter-limb differences in upper quarter mobility/stability was restricted to the YBT–UQ and does not allow statements about other instrumented measures (e.g., maximal shoulder strength or range of motion). Further, the YBT–UQ represents a closed kinetic chain test and thus, our results are not transferable to open chain assessments. Additionally, the preferred swimming styles varied between the groups, which did not allow to calculate style-specific correlations between inter-limb differences in upper quarter mobility/stability and swimming performance. Moreover, our investigation of associations between swimming performance and inter-limb asymmetry was performed using a dry-land test (i.e., YBT–UQ), which limits the transferability of our data to in-water tests (e.g., kinematic and kinetic variables during swimming).

In addition, this study provides some directions for future research. For instance, C-squad compared to A-squad swimmers showed significantly better YBT–UQ performance for the inferolateral reach direction of both arms. Further, significant negative correlations between YBT–UQ and swimming performance were only observed for the B-squad swimmers. Both findings are contrary to our expectations and can only be answered speculatively without further research. Thus, future work should explore the associations between inter-limb differences in upper quarter mobility/stability and athletic performance in bilateral swimming movements but also in mainly unilaterally executed sports (e.g., handball, tennis etc.).

## 5 Conclusions

This work provides insights into how inter-limb differences/performances in upper quarter mobility/stability are related to swimming performance in swimmers with different levels of expertise (A-, B-, and C-squad). Our results showed that YBT–UQ performances (i.e., inferolateral reach direction for both arms) but not inter-limb differences were significantly larger in swimmers with the lowest level of expertise (i.e., C-squad), indicating an optimal ratio of reach distance to arm length for those athletes. We further found that YBT–UQ performances (i.e., inferolateral reach direction and composite score for both arms and medial reach direction for the non-dominant arm) but not inter-limb differences were significantly and negatively correlated with swimming performance among B-squad swimmers. For those athletes, this implies that an increase in upper quarter mobility/stability due to training could a have a detrimental effect on swimming performance.

## Data Availability

The raw data supporting the conclusions of this article will be made available by the authors, without undue reservation.
